# A Comparative Study of EMG Indices in Muscle Fatigue Evaluation Based on Grey Relational Analysis during All-Out Cycling Exercise

**DOI:** 10.1155/2018/9341215

**Published:** 2018-04-16

**Authors:** Lejun Wang, Yuting Wang, Aidi Ma, Guoqiang Ma, Yu Ye, Ruijie Li, Tianfeng Lu

**Affiliations:** ^1^Sport and Health Research Center, Physical Education Department, Tongji University, Shanghai, China; ^2^Physical Education and Sports Science Institute of Shanghai, Shanghai 200030, China

## Abstract

The increased popularization of cycling has brought an increase in cycling-related injuries, which has been suggested to be associated with muscle fatigue. However, it still remains unclear on the utility of different EMG indices in muscle fatigue evaluation induced by cycling exercise. In this study, ten cyclist volunteers performed a 30-second all-out cycling exercise after a warm-up period. Surface electromyography (sEMG) from vastus lateralis muscle (VL) and power output and cadence were recorded and EMG RMS, MF and MPF based on Fourier Transform, MDF and MNF based on wavelet packet transformation, and C(n) based on Lempel–Ziv complexity algorithm were calculated. Utility of the indices was compared based on the grey rational grade of sEMG indices and power output and cadence. The results suggested that MNF derived from wavelet packet transformation was significantly higher than other EMG indices, indicating the potential application for fatigue evaluation induced by all-out cycling exercise.

## 1. Introduction

The increased popularization of cycling in transportation, recreation, and competition has brought an increase in cycling-related injuries [1]. It was found that about 42% to 65% of recreational cyclists may experience overuse knee pain [1, 2]. The incidence of all nontraumatic injuries among cyclists may reach 85% [3]. As muscle fatigue may change the kinematics and muscle activation patterns so as to maintain target performance, the injuries has been suggested to be caused by biomechanical alterations associated with muscle fatigue [4, 5], which indicate that muscle fatigue monitoring and assessment may be helpful in protocol arrangement to reduce injuries during cycling exercise.

Surface electromyography (sEMG) has been widely used in muscle fatigue evaluation due to its noninvasiveness, real time, and applicability [6]. In previous researches, many sEMG indices have been suggested and compared in muscle fatigue assessment, including root mean square (RMS), the median (MF), and mean power frequencies (MPF) based on Fourier Transform [6]. However, these researches have been documented especially for isometric and isokinetic contraction conditions and studies have rarely focused on the EMG-based muscle fatigue evaluation in cycling exercise. As a result, on the utility of different EMG indices in muscle fatigue evaluation induced by cycling exercise still remains unclear.

Muscle fatigue has been defined and quantified by the reduced maximum capacity to generate force or power output [7]. Particularly, the decrease of maximal force or power output has been used as the valid criterion to evaluate the utility of other approaches (e.g., sEMG) in muscle fatigue assessment [8, 9]. In previous researches, the proximity of researched indices changes and the decrease of maximal force or power output has been compared and adopted to evaluate the utility of indices [10], while grey relational analysis is a method to quantifying the proximity of changing trends between inspected sequences and standard sequence by grey relational grade [11, 12]. Therefore, the utility of EMG indices in muscle fatigue can be evaluated by comparing their changing trends with maximum force or power output using grey relational analysis.

This work aims at comparing the utility of sEMG indices in assessing muscle fatigue induced by all-out cycling exercise. EMG indices representing fatigue were calculated and grey rational grade of EMG indices and power output were calculated and compared. The research was performed on the vastus lateralis muscle (VL) as it has been suggested to be the principal power producers during cycling exercise [13, 14]. Results were expected to provide improved EMG-based methods in muscle fatigue assessment for all-out cycling exercise to help reduce injuries of cyclists.

## 2. Materials and Methods

### 2.1. Participants

Seven male and three female cyclist volunteers (age 21.50 ± 4.67 years, height 175.00 ± 8.25 cm, and weight 75.40 ± 10.91 kg) participated in this study, which was approved by the Ethics Committee of Tongji University. The subjects were all healthy, with no known neuromuscular disorders or musculoskeletal injuries, and had not participated in strenuous physical activity in 24 hours before experiment.

### 2.2. Experimental Protocol

The experiment was conducted in laboratory with the indoor temperature of about 24°C and comprised a warm-up exercise and a test exercise. All the exercises were performed on an air-braked ergometer (Wattbike Pro; Wattbike Ltd., Nottingham, United Kingdom) that allows the resistance to be set between 1 and 10 levels. The Wattbike measures the forces applied to the chain over a load cell and angular velocity of the crank twice per revolution to calculate power output at a rate of 100 Hz. Power output and cadence during track cycling were measured using SRM professional power cranks (Schoberer Rad-Messtechnik, Julich, Germany). To ensure accurate measures, a static calibration procedure was conducted before the study for both devices [15].

The warm-up exercise consisted of a 5 min cycling exercise with the air resistance on the ergometer set at level 3 and the cadence at 90 rpm followed by a complete rest of 3 min before the beginning of the test. Based on previous tests with the subjects, the air resistance on the Wattbike ergometer was set to the level at which the subject may produce the maximum power output during all-out cycling exercise. According to this criterion, the air resistance on the ergometer was set to level 10 for seven male cyclists and level 6 for three female cyclists. According to the calibration report, levels 6 and 10 of air resistance on the Wattbike ergometer result in power outputs of 45 and 55 W at a cadence of 40 rpm and 785 and 1045 W at a cadence of 130 rpm. Subjects were asked to produce the highest possible power output for 30 seconds and were verbally encouraged throughout the trials.

### 2.3. EMG Measurement

Surface electromyographic signals were recorded with three round bipolar Ag/AgCl electrodes of ME 6000 P8 Surface EMG acquisition instrument (Mega Electronics System, Finland). Electrodes were placed over the belly of right vastus lateralis (VL) with center-to-center electrode distance setting to 2 cm. The skin was shaved and cleaned with alcohol wipes before the electrodes were fixed. Medical adhesive tape and plastic casts were applied to fix the electrodes. Raw EMG signals were amplified, simultaneously digitized, and acquired by the MegaWin system (Mega Electronics System, Finland) at a sampling rate of 1 kHz.

### 2.4. EMG Data Processing

EMG signals recorded from VL were band-pass filtered at 5–500 Hz using a 4th-order zero-phase-shift Butterworth filter and were divided into every 3-second epochs. For each epoch, EMG RMS, MF (median frequency) and MPF (mean power frequency) based on Fourier Transform, MDF and MNF based on wavelet packet transformation, and C(n) based on Lempel–Ziv complexity algorithm were calculated.

RMS is defined as

(1) $$\begin{array}{c} RMS=\sqrt{\frac{\sum_{i=1}^{n}\left|{\text{rawData}}_{i}\right|}{n}}, \end{array}$$

where *i* represents the order number of the dealing sample point, rawData_*i*_ is the value of the *i*th sample point, and *n* is the total number of the data points.

MF and MPF are defined as follows:

(2) ∫0MFSfdf=∫MF∞Sfdf=12∫0∞Sfdf,MPF=∫0∞Sf·f·df∫0∞Sf·df,

where *f* is the frequency, *S*(*f*) is the power at frequency *f*, and *d*(*f*) is the frequency resolution.

Wavelet packet transformation was employed to analyze sEMG and a wavelet that was a member of the Daubechies family (order 6) was implemented in this analysis. On this basis, MDF and MNF were calculated. MDF and MNF are defined as

(3) ∫0MDFPt,ωdω=∫MDF∞Pt,ωdω=12∫0∞Pt,ωdω,MNF=∫0∞ωPt,ωdω∫0∞Pt,ωdω,

where *P*(*t*, *ω*) represents the power spectrum of EMG signals based on wavelet packet transformation.

Lempel–Ziv complexity was calculated based on complexity C(n) algorithm devised by Kaspar and Schuster [16] and its value is between 0 and 1.

### 2.5. Grey Rational Grade Calculation

In the grey relational grade calculation, EMG indices were selected as inspected sequences while power (or cadence) was chosen as standard sequence. Each data of inspected sequences and standard sequence was normalized by dividing the average value of each sequence. Then the grey relational coefficient was calculated using Deng's grey relational grade formula:

(4) $$\begin{array}{c} \text{corr}\left(\;x_{0}\left(\;k\;\right),x_{i}\left(\;k\;\right)\;\right)=\frac{\Delta min+p\Delta \max}{\Delta_{\text{0}i}\left(k\right)+p\Delta \max}, \end{array}$$

where

1. *i* = 1,2, 3,…, *m*, *k* = 1,2, 3,…, *n*;
2. *x*_0_ is standard sequence and *x*_*i*_ is inspected sequence;
3. Δ_0*i*_ = ‖*x*_0_(*k*) − *x*_*i*_(*k*)‖ is the difference between *x*_0_ and *x*_*i*_;
4. Δ_min._ = ∀_*i*_^min.min.^  ∀*k*‖*x*_0_(*k*) − *x*_*i*_(*k*)‖, Δ_max._ = ∀_*i*_^max.max.^  ∀*k*‖*x*_0_(*k*) − *x*_*i*_(*k*)‖;
5. *p* is distinguishing coefficient, and *p* ∈ [0,1]. In this study, we took *p* = 0.5 according to previous research [11].

When the grey relational coefficient is calculated, the mean value of the grey relational coefficient is taken as the grey relational grade:

(5) $$\begin{array}{c} \text{CORR}\left(\;x_{0},x_{i}\;\right)=\frac{1}{n}\overset{n}{\underset{k=1}{\sum }}\text{corr}\left(\;x_{0}\left(\;k\;\right),x_{i}\left(\;k\;\right)\;\right). \end{array}$$

Grey relational grade of CORR in this study ranged from 0 to 1. A larger value of CORR indicates a more proximity of changing trends between EMG index and power output and thus a better utility to evaluate muscle fatigue.

Data processing was performed using MATLAB R2016a software (Mathworks, USA).

### 2.6. Statistical Analysis

The statistical analysis was performed using SPSS 13.0 for windows (SPSS, Inc., Chicago, IL, USA). Normality was tested using the Kolmogorov-Smirnov test. One-way repeated-measures variance analysis was used to determine the difference of power, cadence, and EMG indices in different pedaling phases. Two-factor variance analysis was used to compare the grey relational grade of different EMG indices and pedaling performance (power and cadence). All significance thresholds were fixed at *α* = 0.05.

## 3. Results

**Figure 1:**
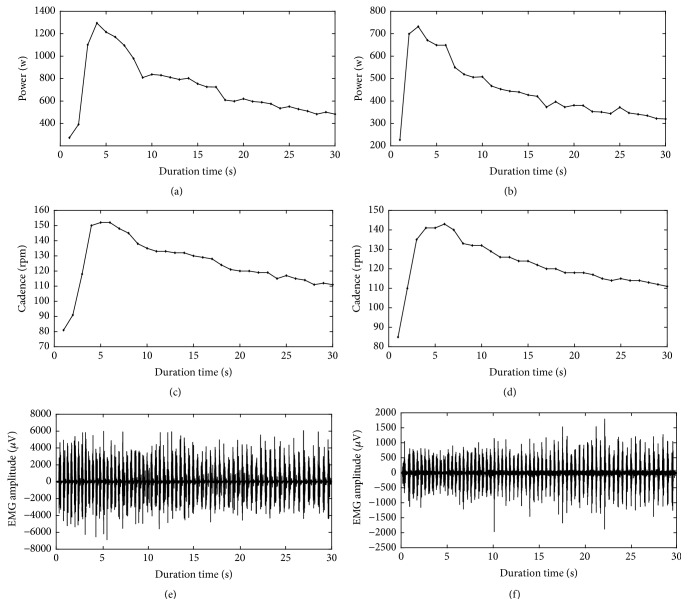
Power output, cadence, and raw EMG signals of vastus lateralis for two representative subjects during 30-second all-out cycling exercise.

Examples of power output, cadence, and raw EMG signals of vastus lateralis are shown in Figure 1. It can be observed from the figure that, during the 30-second all-out cycling exercise, subjects reached their maximum value of power output and cadence in approximately the 4th and 6th second of the exercise, respectively. Power output and cadence began to decrease progressively once they reach the peak value in the later exercise.

**Figure 2:**
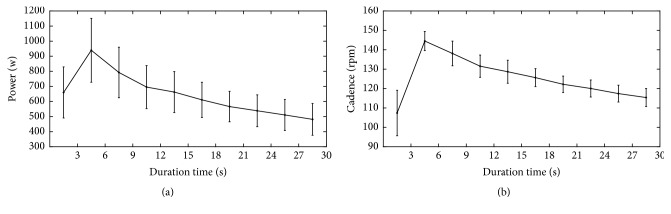
Average power output (a) and pedaling rate (b) of all subjects calculated for every 3-second during cycling exercise.

Figure 2 shows the average power output (a) and cadence (b) of all subjects calculated for every 3-second during cycling exercise. The power reached the maximum value of 939.50 ± 212.06 W in the second 3-second epoch and declined to 481.50 ± 105.56 W in the last 3-second epoch. Correspondingly, the cadence reached 144.50 ± 4.95 RPM in the second 3-second epoch and decreased to 115.37 ± 4.63 RPM in the last 3-second epoch. One-way repeated-measures variance analysis results revealed that power output and cadence had significant difference in 10 pedaling periods (power: *F* = 32.858, *P* ≤ 0.001; cadence: *F* = 46.705, *P* ≤ 0.001). Power output and cadence showed a progressively approximate linear decrease tendency from the third 3-second cycling exercise.

**Figure 3:**
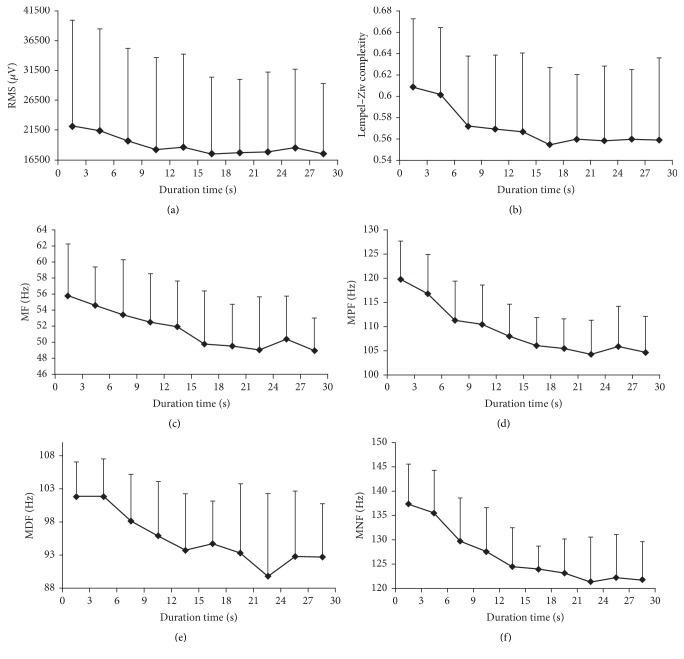
Average EMG RMS, C(n), MF, MPF, MDF, and MNF of all subjects calculated for every 3-second during cycling exercise.

Figure 3 showed the average EMG RMS, C(n), MF, MPF, MDF, and MNF of all subjects calculated for every 3-second during cycling exercise. The RMS, C(n), MF, MPF, MDF, and MNF in the first and last 3-second epoch were 22126.89 ± 17805.51 *μ*V, 55.80 ± 6.45 Hz, 119.82 ± 7.77 Hz, 101.83 ± 5.23 Hz, 137.49 ± 8.02 Hz, 0.61 ± 0.06 and 17635.63 ± 11687.06 *μ*V, 48.95 ± 4.06 Hz, 104.73 ± 7.37 Hz, 92.72 ± 8.05 Hz, 121.79 ± 7.82 Hz, and 0.56 ± 0.08, respectively. During the pedaling exercise, the EMG RMS, C(n), MF, MPF, MDF, and MNF all showed a declining tendency while EMG MF, MPF, MDF, and MNF showed a more significant decreased tendency compared with EMG RMS and C(n). One-way repeated-measures variance analysis results revealed that the duration time factor had significant influence on EMG RMS, MF, MPF, MDF, MNF, and C(n) (RMS: *F* = 2.957, *P* ≤ 0.05; MF: *F* = 6.315, *P* ≤ 0.001; MPF: *F* = 13.114, *P* ≤ 0.001; MDF: *F* = 3.969, *P* ≤ 0.001; MNF: *F* = 8.915, *P* ≤ 0.001; C(n): *F* = 6.087, *P* ≤ 0.001). Post hoc analysis showed that most of MF, MPF, MDF, and MNF values were significantly decreased compared to the value of precedent.

Table 1 shows the grey relational grade between EMG indices and pedaling performance. The grey relational grades between EMG indices of RMS, MF, MPF, MDF, MNF, C(n), and power were 0.47 ± 0.06, 0.70 ± 0.06, 0.71 ± 0.03, 0.68 ± 0.06, 0.78 ± 0.05, and 0.56 ± 0.09, while the grey relational grades between EMG indices and cadence were 0.43 ± 0.09, 0.70 ± 0.06, 0.69 ± 0.06, 0.67 ± 0.08, 0.74 ± 0.05, and 0.47 ± 0.07.

The statistical analysis revealed significant effects of both inspected sequences (*F* = 68.241, *P* ≤ 0.001) and standard sequences (*F* = 7.348, *P* ≤ 0.01) on the grey relational grade value. No significant interaction effect of inspected sequences by standard sequences was found (*F* = 1.207, *P* > 0.05). Post hoc analysis showed that grey relational grade calculated between EMG indices and power was significantly greater than between cadence. On the other hand, grey relational grade of MNF was significantly higher than other EMG indices, while grey relational grade of RMS and C(n) was significantly lower than other four EMG indices (*P* < 0.05).

## 4. Discussion

The main purpose of this study was to determine the utility of several EMG-based fatigue indices in the context of cycling exercise. In the comparative research of EMG-based muscle fatigue evaluation, previous studies have mainly focused on isometric and isokinetic contraction conditions and rarely have focused on the cycling exercise [6, 10, 17, 18]. In the present study, the utility of the EMG indices in muscle fatigue evaluation was quantified based on the grey rational grade between EMG indices and power output. Results of this study suggested that grey relational grade of MNF was significantly higher than other EMG indices, indicating the potential application for fatigue evaluation induced by all-out cycling exercise.

In the previous research [19], we have compared sensitivity and stability of EMG RMS, MPF, MNF, and C(n) in evaluation of rectus femoris fatigue induced by 60-second all-out cycling exercise and found that MNF have the highest fatigue sensitivity while RMS had the lowest fatigue sensitivity. The sensitivity of MPF and C(n) had no significant difference. Referring to stability, C(n) was the optimum index and MNF the second, followed by MPF and RMS. In this study, the optimum utility of MNF and inferior utility of RMS have also been found, which is consistent with the previous research. However, C(n) was not found to have favourable advantage compared to MF and MPF.

RMS showed inferior utility compared to other indices, indicating inconsistent performance to evaluate muscle fatigue. The result is in agreement with previous studies which have revealed the inconsistent changes of RMS during contractions [20]. As RMS can be easily influenced by experiment conditions such as muscle contraction style, workload, endurance time, and other factors, the use of this index as fatigue indicator should be interpreted with caution [10, 21].

MF and MPF have been accepted as the representative indicators of muscle fatigue and have been widely employed in muscle fatigue evaluation in previous researches [22, 23]. It has been reported that, both in isometric and in dynamic fatiguing contraction conditions, MF and MPF showed significant decreasing tendency [1, 24, 25]. However, other studies have shown concern on the use of MF and MPF as fatigue index in dynamic contraction due to the limitation of Fourier Transform in nonstationary signal analysis [26]. In this study, EMG recorded from VL were nonstationary and nonlinear signals due to the obvious electrode shifts relative to the muscle fiber and changes in the conductivity property of the tissues in the strenuous all-out cycling exercise, which may influence the sensitivity and stability of MF and MPF as fatigue indices [27–29]. The results showed that the grey relational grade of MF and MPF were higher than RMS and C(n) and lower than MNF, indicating that MF and MPF were not the optimum fatigue indices in all-out cycling exercise.

Wavelet transformation has been suggested to be suitable for the analysis of nonstationary myoelectric signals recorded in dynamic contractions. Karlsson et al. [30] have shown that continuous wavelet transform has better accuracy in estimating time-dependent spectral moments than those obtained by using short-time Fourier Transform, the Wigner–Ville distribution, and the Choi–Williams distribution methods [13]. Karlsson et al. used wavelet transformation to study movements at different angular velocities and found that wavelet transform was very reliable for the analysis of nonstationary biological signals [31]. In agreement with previous researches, the grey relational grade of MNF was significantly higher than other parameters, indicating the optimum utility in muscle fatigue assessment.

Lempel–Ziv complexity was calculated using regression function method and can reflect new pattern generating speed of time series along with its length increase and reflects the randomness of the time sequence [32]. Previous researches have demonstrated that the Lempel–Ziv complexity method is easy to use and effective in revealing the dynamic characteristics and variations of exercise fatigue [33, 34]. In our previous research, Lempel–Ziv complexity showed the highest stability while the sensitivity was lower than MPF and MNF [19]. Therefore, the poor utility of Lempel–Ziv complexity C(n) revealed in this study may attribute to the low sensitivity of reflecting muscle fatigue and is not suitable for the fatigue index induced by cycling exercise.

Muscle fatigue represents a complex phenomenon and encompasses a number of changes occurring at both central and peripheral level [35–38]. Although central and peripheral mechanisms are highly interactive, the contribution of two mechanisms may be quite different in certain conditions. For example, previous researches have demonstrated that muscle fatigue induced by very high force level mainly occurred at perpetual level while sustained contraction at low forces produces prominent central fatigue [39–42]. In this study, subjects performed very vigorous dynamic cycling exercise with high force level and no doubt would lead to remarkable perpetual fatigue as well as central fatigue. As utility of EMG indices may be quite different in evaluating central fatigue and peripheral fatigue [39–42], the difference utility of EMG variables in muscle fatigue evaluation would be explained by the different fatigue mechanism of central and perpetual factors.

In conclusion, surface EMG recorded from VL during all-out cycling exercise was nonlinear and nonstationary signals that refrain the application of Fourier Transform, while MNF derived from wavelet packet transformation showed the maximum grey rational grade with both power output and cadence, indicating the potential application for fatigue evaluation induced by all-out cycling exercise.

## Figures and Tables

**Table 1:** Grey relational grade between EMG indices and pedaling performance.

	RMS	MF	MPF	MDF	MNF	C(n)
Power	0.47 ± 0.06	0.70 ± 0.06	0.71 ± 0.03	0.68 ± 0.06	0.78 ± 0.05	0.56 ± 0.09
Cadence	0.43 ± 0.09	0.70 ± 0.06	0.69 ± 0.06	0.67 ± 0.08	0.74 ± 0.05	0.47 ± 0.07
